# Atherosclerosis and Atheroma Plaque Rupture: Imaging Modalities in the Visualization of Vasa Vasorum and Atherosclerotic Plaques

**DOI:** 10.1155/2014/312764

**Published:** 2014-02-11

**Authors:** Zhonghua Sun

**Affiliations:** Discipline of Medical Imaging, Department of Imaging and Applied Physics, Curtin University, P.O. Box U1987, Perth, WA 6845, Australia

## Abstract

Invasive angiography has been widely accepted as the gold standard to diagnose cardiovascular pathologies. Despite its superior resolution of demonstrating atherosclerotic plaque in terms of degree of lumen stenosis, the morphological assessment for the plaque is insufficient for the analysis of plaque components, and therefore, unable to predict the risk status or vulnerability of atherosclerotic plaque. There is an increased body of evidence to show that the vasa vasorum play an important role in the initiation, progression, and complications of atherosclerotic plaque leading to major adverse cardiac events. This paper provides an overview of the evidence-based reviews of various imaging modalities with regard to their potential value for comprehensive characterization of the composition, burden, and neovascularization of atherosclerotic plaque.

## 1. Introduction

The development of atherosclerosis is associated with structural and functional changes in the vascular wall [1]. Adventitial vasa vasorum are functional endarteries that are normally present on the adventitial side of the arteries [2, 3]. The main functions of the vasa vasorum are to deliver nutrients to the vessel wall and to remove waste products or noxious substances [4]. The density of this vascular network increases comparably with the natural growth of the vessel wall and is closely associated with the progression of vessel wall disease, particularly those diseases involving inflammation, including atherosclerosis and diabetes [5].

Adventitial vasa vasorum has been shown to participate in the process of atherogenesis and atherosclerotic plaque progression [6–8]. Extensive research has been conducted on experimental animals and human autopsy data in the past 20 years to support the hypothesis that vasa vasorum-derived plaque neovascularization is intimately associated with atherosclerotic plaque progression and destabilization.

Vasa vasorum can be visualized directly or indirectly by several imaging modalities in experimental models and clinical settings, including microcomputed tomography (CT), contrast-enhanced ultrasound (CEUS), intravascular ultrasound (IVUS), optical coherence tomography (OCT), and contrast-enhanced magnetic resonance imaging (CE-MRI). These imaging techniques provide a unique opportunity to demonstrate the normal anatomy of vasa vasorum and monitor progressive pathophysiological developments associated with atherosclerosis due to vasa vasorum injury. This review paper focuses on different imaging modalities for identification and visualization of vasa vasorum and neovascularization, as well as characterization of plaque components.

## 2. Imaging Modalities in the Visualization of Vasa Vasorum and Atherosclerotic Plaques

### 2.1. Micro-CT

Micro-CT is an ex vivo imaging modality capable of achieving a spatial resolution on the order of 20 *μ*m^3^, which enables acquisition of high spatial resolution images of vasa vasorum in human and animal models [9, 10]. A micro-CT scanner generates 3D images consisting of up to a billion cubic voxels, with each 5–25 *μ*m on a side having isotropic spatial resolution. Its main components include a spectroscopic X-ray source that produces selectable primary emission peaks at ~9, 18, or 25 keV and a fluorescing thin crystal plate that is imaged with a lens onto a 2.5 × 2.5-cm, 1024 × 1024-pixel charge-coupled device (CCD) detector array [11]. The specimen is positioned close to the crystal and is rotated around 360° between each X-ray exposure and its CCD recording, with tomographic reconstruction algorithms applied to these recorded images, which are used to generate 3D images of the specimen. In order to differentiate grayscale values in the media and adventitia area in arteries, the voxel sizes should be smaller than 200 *μ*m^3^ to allow quantitative analysis of vasa vasorum in the coronary wall [12]. The latest multislice CT scanners provide voxel sizes greater than 350 *μ*m^3^ [13], which make it difficult to visualize the vasa vasorum.

Kwon et al. in their early study showed the 3D anatomy of vasa vasorum in both normal and balloon-injured coronary arteries [14]. In their study, the first-order vasa vasorum arising from the lumen of the coronary artery which ran longitudinally along the media-adventitial layer and the smaller second-order vasa vasorum arising from branches of first-order vasa vasorum were clearly demonstrated in normal pig arteries. Their results showed that the mean diameter of normal first-order vasa vasorum was 160.92 ± 5.10 *μ*m and of second-order vasa vasorum, 67.99 ± 2.72 *μ*m (*P* < 0.0001), while in the injured coronary arteries, the mean diameter of first- and second-order vasa vasorum was 141.11 ± 5.87 *μ*m and 101.59 ± 1.49 *μ*m, respectively, with significant differences between normal and injured arteries (*P* < 0.05). In addition, marked adventitial neovascularization was observed in the injured porcine coronary arteries when compared to the normal coronary arteries in terms of vessel wall area (4.85 ± 0.35 mm^2^ versus 11.24 ± 0.32 mm^2^, *P* = 0.0001, resp.) and density of vasa vasorum (3.16 ± 0.17 per mm^2^ versus 1.90 ± 0.06 per mm^2^, *P* = 0.0001). Later reports using micro-CT further confirmed the clinical value of this high-resolution noninvasive imaging modality in terms of quantitative assessment of vasa vasorum distribution in the coronary arteries and its role in the development of atherosclerosis [15–21].

There is a growing body of evidence showing that vasa vasorum neovascularization plays an important role in the progression and complications of atherosclerosis. In an experimental hypercholesterolemia study, Herrmann et al. used 3D micro-CT to demonstrate an increase in coronary vasa vasorum density within the first 4 weeks prior to the development of endothelial dysfunction, therefore, suggesting a role for vasa vasorum neovascularization in the initiation of atherosclerosis [15]. The density of vasa vasorum was significantly increased in animals on a hypercholesterolemic diet for 2 and 4 weeks (4.88 ± 2.45 per mm^2^) and 6 and 12 weeks (4.50 ± 1.37 per mm^2^) when compared to the control group (2.97 ± 1.37 per mm^2^). This is confirmed by experimental studies using antiangiogenic drug to inhibit inflammatory effects, consequently inhibiting vasa vasorum neovascularization [16–19]. Gössl et al. used micro-CT to compare and analyse vasa vasorum density in normal and high-cholesterol diet animals [17]. They reported that vasa vasorum density and intima-media thickness were significantly increased in the hypercholesterolemic animals when compared to the normal group (6.4 ± 0.7 mm^2^ versus 2.7 ± 0.3 mm^2^, 0.62 ± 0.09 mm versus 0.28 ± 0.02 mm, corresponding to vasa vasorum density and intima-media thickness, resp., *P* < 0.05). Furthermore, prevention of vasa vasorum neovascularization and significant reduction of vascular endothelial growth factor were achieved through administration of anti-angiogenic drug, which led to a significant reduction of vascular area fraction, intima-media thickness, and endothelial exchange surface within the coronary artery wall [4, 17–19]. These observations contribute to the inhibition of neointima proliferation, which in turn inhibits the development of early atherosclerosis [20–22].

**Figure 1:**
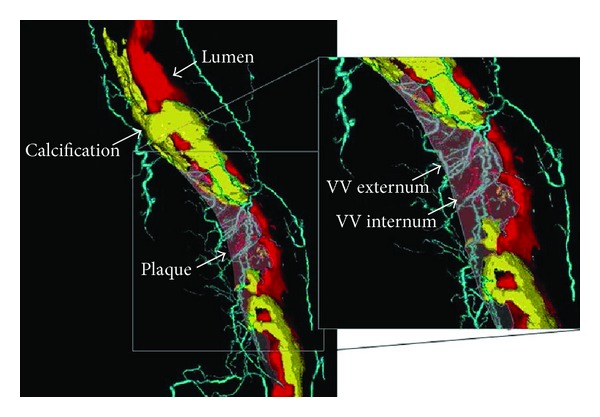
Volume-rendered micro-CT imaging of a coronary plaque and vasa vasorum. Volume-rendered 3D micro-CT image of a right coronary artery showing the main coronary lumen in red; noncalcified and calcified plaque areas are indicated by the arrows (noncalcified plaque is transparent). Vasa vasorum (VV) are shown in light blue (VV externa) or red (VV interna, directly originating from the main lumen). Reprint with permission from [23].

In addition to visualization of normal anatomy of vasa vasorum distribution and pathological change along the artery wall, micro-CT is also able to detect segmental vasa vasorum neovascularization in the coronary artery. Based on 20 coronary segments from 15 autopsy patients, Gössl and colleagues in their study found that vasa vasorum density was higher in artery segments with nonstenotic plaques and with noncalcified stenotic plaques than that in the normal segments (3.36 ± 0.45 mm^2^, 3.73 ± 1.03 mm^2^ versus 1.16 ± 0.21 mm^2^, *P* < 0.01), while in the presence of significant calcification, vasa vasorum spatial density decreased significantly to the levels similar to those observed from normal coronary segments (0.95 ± 0.21 mm^2^) (Figure 1) [23]. Coronary calcification is regarded as a reliable indicator of atherosclerosis and its extent is related to the plaque burden, but not to the degree of obstruction [24, 25]. However, the mechanism of the calcification process, whether it is part of the inflammatory damage or part of the repair process, remains to be understood [26].

In summary, micro-CT has demonstrated superior spatial resolution which allows for quantitative imaging of the spatial and temporal distribution of vasa vasorum in the aorta and coronary artery [15, 17, 23, 27]. Vasa vasorum parameters including vasa vasorum count, vasa vasorum spatial density, vasa vasorum vascular area fraction, and vasa vasorum endothelial surface fraction can be reliably evaluated with use of micro-CT [18, 26, 27]. The increase in arterial wall opacity due to increased vascularity of the vasa vasorum can also be detected on micro-CT images, and this serves as an index of the angiogenesis that takes places in early plaque development [28]. These research findings corroborate the clinical value of micro-CT imaging in the investigation of atherosclerosis due to vasa vasorum injury and its role in the initiation and progression of atherosclerotic plaques.

### 2.2. Contrast-Enhanced Ultrasound

Recently, ultrasound contrast agents have been developed and used to visualize the carotid artery vasa vasorum and neovascularization of atherosclerotic plaques. Contrast-enhanced ultrasound (CEUS) is a promising noninvasive modality for visualization of plaque neovascularization [29]. Microbubble ultrasound contrast agents are confirmed to be clinically useful in enhancing ultrasound images and improving the diagnostic accuracy [30]. The real-time CEUS imaging enables a dynamic evaluation of microvascular assessment of the spatial and temporal heterogeneity of adventitial and intraplaque vasa vasorum [29–32]. The usefulness and reliability of CEUS have been validated by previous studies in animals and humans that showed the degree of plaque neovascularization which is correlated closely with density of neovessels [33–35].

**Figure 2:**
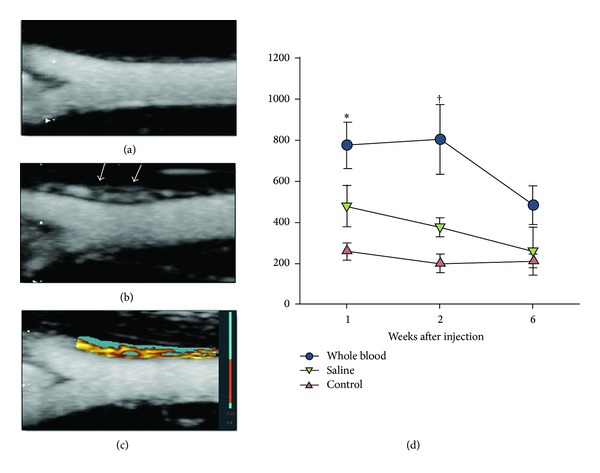
Vasa vasorum blood volume on contrast-enhanced ultrasound imaging. Examples of maximum intensity projection images 2 s after the destructive pulse sequence are shown for femoral arteries 2 weeks after injection of either saline (a) or whole blood (b), illustrating a greater vasa vasorum (VV) density (arrows) in the latter. (c) Example of pixel intensity threshold analysis for the blood-injected vessel whereby pixels within the region of interest that enhance beyond threshold intensity are displayed in red-orange color scale and those that do not are displayed in blue. (d) Mean (± standard error of the mean) area of enhancement on pixel intensity threshold analysis, an index of functional VV blood density. Data for contralateral noninjected control vessels were similar between treatment cohorts and are grouped. *P < 0.05 versus control contralateral artery; ^†^ *P* < 0.05 versus both contralateral and saline-injected arteries (corrected for multiple comparisons). Reprint with permission from [41].

Schinkel et al. demonstrated the feasibility of imaging vasa vasorum using CEUS in their animal experiments. CEUS is able to detect and monitor the progression of intima-media thickness and the density of the vasa vasorum network in the follow-up of swine models of atherosclerosis (0.22 ± 0.05 mm versus 0.45 ± 0.06 mm at baseline, *P* < 0.001) (Figure 2) [36]. Clinical reports on patient data further corroborated the feasibility of using CEUS in the visualization of adventitial neovascularization. Giannoni et al. in their pilot study showed that CEUS has potential value in imaging plaque neovascularization (73 plaque specimens) between symptomatic and asymptomatic patients. In patients with acute symptomatic disease, CEUS detected a specific pattern of diffuse contrast enhancement at the base of the carotid plaques, close to the adventitial layer of the artery wall. This corresponded to a high density of small diameter microvessels, indicating the presence of plaque angiogenesis, despite inclusion of only 9 symptomatic patients in their group [37]. Their findings are further validated by Xiong et al.′s study (133 plaques) which involved comparing 35 symptomatic patients with 69 asymptomatic patients using CEUS [38]. A higher prevalence of carotid plaque enhancement was observed in symptomatic patients than in the asymptomatic group, showing good correlation between the degree of contrast enhancement and patient symptoms. Their results showed that enhanced intensity in the plaque (13.9  ± 6.4 dB) and the ratio of enhanced intensity in the plaque to that in the lumen of the carotid artery (0.54 ± 0.23) in symptomatic patients were significantly higher than those in asymptomatic patients (8.8  ± 5.2 dB and 0.33 ± 0.19, *P* < 0.001). These findings confirm that CEUS may be used for plaque risk stratification and for the assessment of progression of atherosclerosis [38–40].

Traditional CEUS has limited value in quantitative assessment of progression or regression of vasa vasorum due to the effect of microbubble concentration in the blood pool, which is variable over time and between subjects. Lee et al. in their recent study used maximum intensity projection (MIP) processing of CEUS images to enhance diagnostic sensitivity for detection of microvessels in vitro animal studies [41]. Their MIP technique allows for detection of microvascular linear intensity by tracking the entire course of microbubble transit through a microvessel, with a 3-fold increase in femoral artery vasa vasorum microvascular density after blood injection compared to the saline-exposed group (201 ± 11 vessels versus 76 ± 10 vessels per section, *P* < 0.05). Therefore, CEUS with MIP represents an advantage over conventional methods by providing higher average intensities with data less affected by microbubble concentration. The presence of microvessel in the femoral artery adventitia could be detected at 2 weeks using CEUS, indicating the improved accuracy of CEUS with MIP processing for evaluation of vasa vasorum functional density.

In summary, CEUS represents a new opportunity for noninvasive in vivo imaging of atherosclerotic plaques, and its clinical value has been confirmed in both animal experiments and human studies to be a feasible imaging method for visualization of adventitial vasa vasorum neovascularization. As shown in the above-mentioned studies, there is a positive correlation between vasa vasorum density and atherosclerosis and plaque progression. Furthermore, CEUS demonstrates that vasa vasorum was inhibited due to use of antiatherosclerotic drugs, with the development of atherosclerosis being delayed [42]. However, differentiation of stable from unstable plaques by CEUS could be difficult as shown in a recent study by Vavuranakis et al. [43]. CEUS showed the increase in brightness in carotid plaques which correlated with plaque neovessels for stable plaques, but this was not observed in unstable plaques, although more contrast enhancement would be expected to be present in unstable plaque reflecting a more pronounced vascularization. Therefore, further studies are needed to verify the presence of vasa vasorum in atherosclerotic plaques, particularly focusing on the unstable or vulnerable plaques.

### 2.3. Intravascular Ultrasound

Intravascular ultrasound (IVUS) provides high-resolution tomographic images of the lumen and it is a widely used method for assessing atherosclerotic coronary lesions, guiding stent deployments [44, 45]. IVUS can be used to acquire precise and reproducible measurements of atherosclerotic plaques in vivo and serially assess the effects of pharmacological treatment on plaque over time in human and animal studies [46]. IVUS imaging systems which are developed to examine flow within the lumen of large arteries are not able to detect vasa vasorum [47]. Using contrast agents has been reported to induce IVUS echogenicity enhancement in the adventitia of coronary arteries, consistent with the detection of vasa vasorum [48]. Studies on animal experiments and human subjects demonstrated the potential of evaluating vasa vasorum in atherosclerotic plaques using IVUS.

Goertz et al. demonstrated the feasibility of using contrast-enhanced IVUS for visualization of vasa vasorum in phantom and vivo experiments [49, 50]. They used the harmonic contrast imaging method to detect microvascular flow so as to assess the small adventitial vessels consistent with the detection of vasa vasorum. Moritz et al. in a porcine model showed that IVUS allowed for assessment of the density of vasa vasorum [51]. Quantification of the total vasa vasorum flow was performed in their study by summing the blood flow within the vasa vasorum using IVUS, and this was comparable to the quantification of the 3D distribution of vasa vasorum assessed by micro-CT. Their results demonstrated a high and significant correlation between IVUS and micro-CT in vivo in the visualization of vasa vasorum, which is an indicator of atherosclerotic changes.

**Figure 3:**
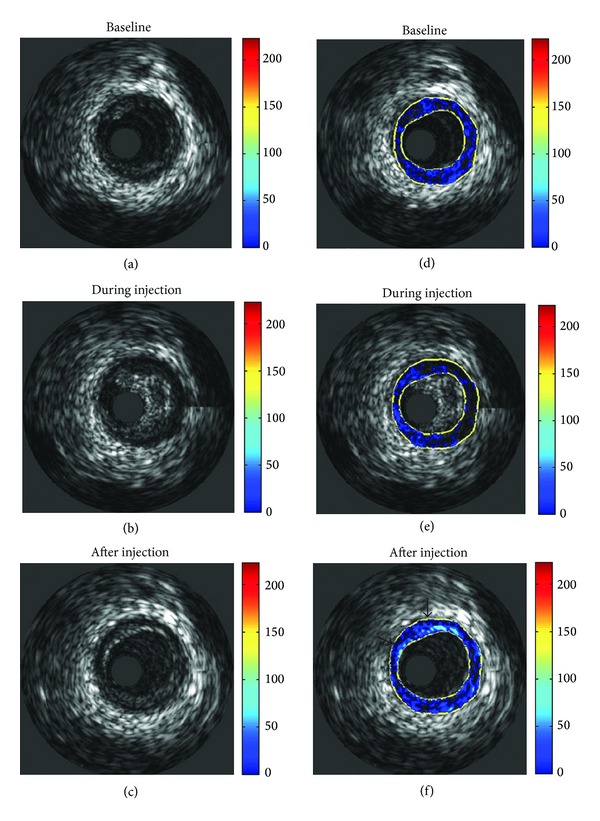
Depiction of qualitative representation of enhancement. Unprocessed images are displayed (a) before, (b) during, and (c) after injection of microbubbles. Corresponding processed images are displayed in (d)–(f). Enhancement is graded from minimal (blue) to maximal (red). Values are a percentage of the maximum grey level intensity difference (255). Arrows indicate points of intense, stable enhancement at the media-adventitia border. Diffuse points of enhancement are present nearby. Reprint with permission from [53].

Clinical studies on patients further confirmed these experimental results. O'Malley et al. used microbubble contrast-enhanced IVUS in 7 patients with coronary artery disease to image and analyse the density and perfusion of vasa vasorum in atherosclerotic plaques [52]. Their results, for the first time, represented the in vivo imaging of vasa vasorum by IVUS. Vavuranakis et al. in their clinical study consisting of 16 patients with acute coronary syndrome validated the feasibility of detecting microbubbles in the coronary lumen using contrast-enhanced IVUS (Figure 3) [53]. The authors reported that contrast-enhanced IVUS enabled quantitative analysis of the echogenicity of the intima-media, adventitia and combined intima-media and adventitia with significant enhancement observed following injection of contrast medium (intima-media: from 6.0 ± 2.5 to 7.9 ± 3.3 mm, *P* = 0.006 and adventitia: from 7.1 ± 2.2 to 7.6 ± 2.5 mm, *P* = 0.035). These changes are consistent with vasa vasorum microvessels, which are characteristic features of vulnerable plaques.

In summary, IVUS is a promising technique for detecting the increased volume of blood flow in the vasa vasorum, which forms a basis for analysing atherosclerotic plaques. Although IVUS provides important information about changes in the material content in the arterial wall, it has limited value in quantifying the density of vasa vasorum in the arterial wall. Combined CEUS and IVUS may enhance the diagnostic value of IVUS by providing an opportunity to obtain more direct information in the vascular biology, as shown in a recent study [42].

### 2.4. Optical Coherence Tomography

Optical coherence tomography (OCT) is a recently developed intravascular imaging modality using near-infrared light to generate cross-sectional intravascular images [54–57]. The imaging principle of OCT is that the electric field amplitude of light reflected from the sample at a certain depth is measured using the principle of low coherence interferometry, with a short coherence length of the source of radiation [58, 59]. The intensity of the interferometric signal is converted to a color-scale or gray-scale to produce cross-sectional images of tissue sample. There are two types of OCT systems that are available to provide intravascular images: first generation OCT systems, known as Time Domain (TD) OCT [58, 59], and second generation systems, known as Fourier Domain (FD) [60, 61]. TD-OCT uses a broadband light source in the range between 1280 and 1350 nm band to perform multiple scanning of reference delay distance and directly measure the electric field amplitude. By contrast, FD-OCT uses a monochromatic laser with wavelength changing over time, while the reference delay distance remains constant, and the electric field amplitude is computed through Fourier transformation and is detected at all depth points simultaneously.

The greatest advantage of OCT is its high resolution (10–20 *μ*m), which is 10 times higher than that of IVUS and is comparable to that of micro-CT. OCT can differentiate three layers of the artery wall by showing the adventitia as signal rich layer surrounding the signal poor layer of the media and signal rich layer of the intima closest to the lumen [62, 63]. OCT also allows tissue characterization by identifying three types of plaques, such as fibrous, fibrocalcific, and lipid; therefore, OCT is regarded as a suitable imaging modality for quantifying the thickness of thin cap fibroatheroma and estimating macrophage distribution (vulnerable plaques) [64–66]. OCT has the ability to characterize these microscopic features of vulnerable plaques which makes it a unique imaging modality.

**Figure 4:**
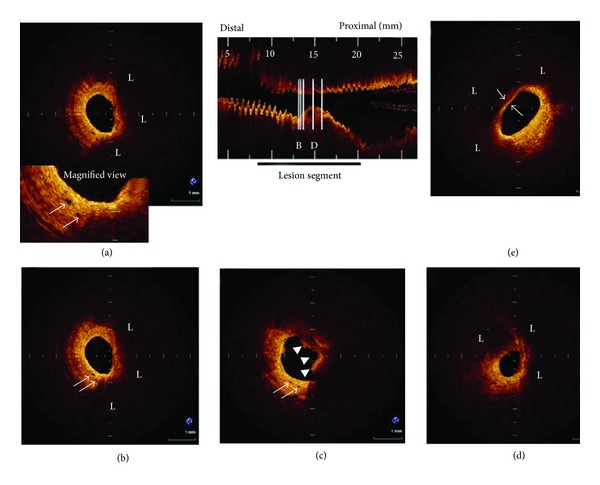
Representative OCT images of coronary plaque with microchannels. (a), (b) Two consecutive cross-sectional OCT images. Eccentric lipid-rich plaque (L) was imaged. Two microchannels (arrow) were located in thickened intima at shoulder region of plaque. (c) Proximal site adjacent to plaque imaged in Figures (a) and (b). Two microchannels (arrow) were located in thickened intima at 7-o'clock position. Intracoronary thrombus (arrowhead) was also visualized. (d) Minimum lumen area site. Lipid-rich plaque was visualized. (e) Lipid-rich plaque covered by thin fibrous cap (50 *μ*m) imaged and found to be thin cap fibroatheroma. Reprint with permission from [69].

Following the successful first application of OCT in 10 patients for assessment of plaque vulnerability [67], a number of clinical studies have been performed to evaluate the association between vasa vasorum and atherosclerotic plaques, in particular, the vulnerable plaques using OCT technique. Yabushita et al. in their vitro study examined 357 diseased atherosclerotic arterial segments with results compared to histologic examination [68]. OCT was found to have sensitivity and specificity of 71% to 79% and 97% to 98% for fibrous plaques, 95% to 96% and 97% for fibrocalcific plaques, and 90% to 94.5% and 90% to 92% for lipid-rich plaques, respectively. Kitabata et al. showed that microchannels were detected in 38% of culprit plaques using OCT, with a significant difference observed in the frequency of thin cap fibroatheroma between patients with and without microchannels (54% versus 21%, *P* = 0.012) (Figure 4) [69]. In addition, the thickness of the fibrous cap was significantly thinner in the patients with microchannels (60 *μ*m versus 100 *μ*m, *P* = 0.001). Other studies further confirmed the potential value of OCT in evaluating vulnerable plaques in patients with coronary artery disease based on short-to long-term follow-up.

Uemura et al. used OCT to analyse 53 patients with coronary artery disease consisting of 69 nonsignificant coronary stenosis plaques (NSCPs) in terms of plaque characteristics and plaque progression at a mean follow-up of 7 months [70]. Their results showed a significantly higher incidence of intimal laceration (61.5% versus 8.9%), microchannels (76.9% versus 14.3%), lipid pools (100% versus 60.7%), thin cap fibroatheroma (76.9% versus 14.3%), macrophage image (61.6% versus 14.3%), and intraluminal thrombus (30.8% versus 1.8%) in NSCPs with progression than those with NSCPs without progression (*P* < 0.05 for all of these comparisons). Barlis et al. showed that OCT could be safely used in vivo to show the culprit coronary lesions and detect morphologic features associated with plaque vulnerability [71]. During 24 months of follow-up of 23 patients prior to coronary angioplasty, OCT detected 7 thin cap fibroatheroma lesions in 6 patients with a mean cap thickness of 0.19 ± 0.05 mm. Their results together with others indicated that OCT is a valuable technique for assessing culprit atherosclerotic lesions in vivo with favourable results achieved [72, 73].

One important aspect about vasa vasorum is that they are dynamic, and the blood flow in these structures can be detected by OCT technique. Cheng and colleagues developed an intensity kurtosis OCT technique to visualize vasa vasorum of carotid artery in vivo [74]. The filling of blood into the vasa vasorum and dynamic motions of the arterial wall were clearly demonstrated using their OCT technique. Their method may provide useful information for evaluating the health status of the artery through imaging of vasa vasorum and associated abnormal changes such as proliferation, thus, improving treatment of atherosclerosis in coronary and carotid arteries, although further research is required to verify their early results.

In summary, OCT is an exciting light-based imaging modality with excellent spatial resolution and a strong contrast between the lumen and artery wall structure [75, 76]. OCT has been validated in both in vitro and in vivo studies to characterize plaque components and identify microvessels and vulnerable plaques which are seen with thin fibrous cap and increased neovascularization of atherosclerosis [70–73, 77–79]. OCT suffers from some limitations. It is not widely available in many catheterization centres. Another main limitation of OCT is the limited depth penetration through tissues, which is less than 2 mm. This will significantly affect the role of OCT to assess plaque burden [56]. Quantitative analysis of subtle morphological parameters such as fibrous cap thickness or compositional parameters could be challenging and may need trained reader interpretation [80]. Furthermore, OCT has been reported to have a moderate diagnostic value in identifying hemodynamically significant coronary stenosis when compared with fractional flow reserve and IVUS [81]. Recently, international guidelines have been developed by the International Working Group for Intravascular OCT Standardization and Validation (IWG-IVOCT) [82]. The document is recommended for being broadly used as a standard reference regarding the current state of the IVOCT imaging modality in clinical practice.

### 2.5. Contrast-Enhanced MRI

MRI is currently recognized as one of the most valuable imaging modalities for the quantification of vascular plaque burden and assessment of atherosclerotic plaque composition [83–85]. It is well understood that neovascularization of the vessel wall plays a key role in atherosclerotic plaque development and progression; thus, increased neovascularization is often associated with markers of plaque vulnerability such as intraplaque hemorrhage and thin cap fibroatheroma [86, 87]. Neovessels arising from the vasa vasorum provide nutrients to thickening walls and serve as an entry site for inflammatory cells into the plaque [88, 89]. Thus, the extent of vasa vasorum may be associated with atherosclerotic plaque size, vascularity, composition, and inflammation [90].

Several different MRI techniques have been developed to assess plaque neovascularization in recent years, and dynamic contrast-enhanced MRI (DCE-MRI) represents a promising technique as it allows for the assessment of neovascular architecture and functional characteristics such as fractional plasma volume and permeability [92, 93]. DCE-MRI also offers high spatial resolution that enables localization of the adventitial boundary, which is the source of the vasa vasorum [94, 95].

**Figure 5:**
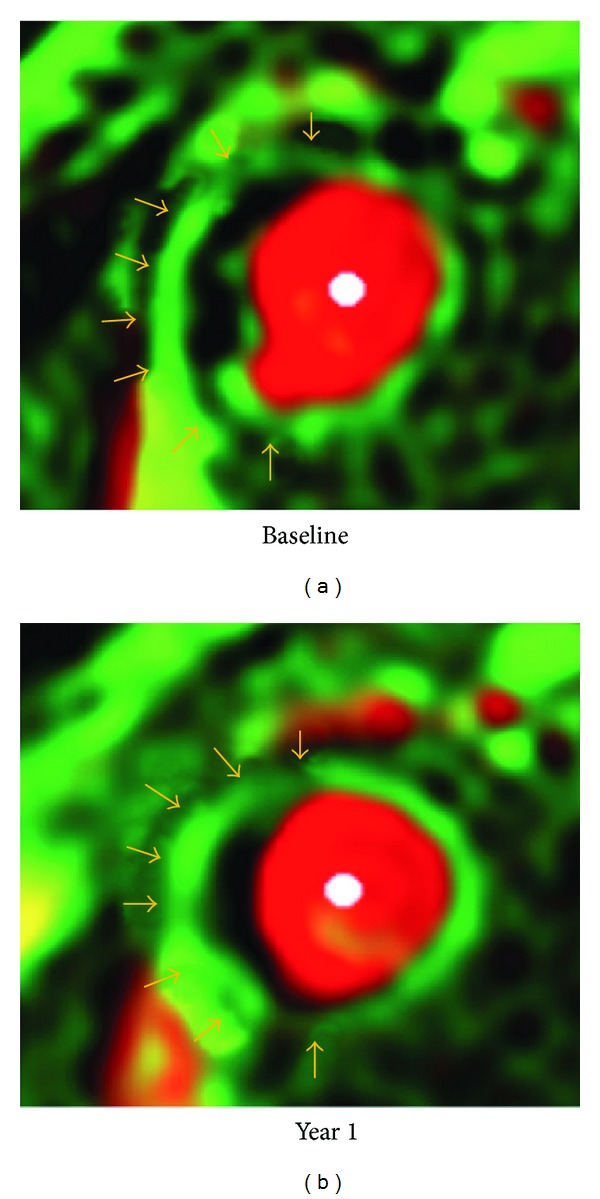
Vasa vasorum images in a 63-year-old man with hypertension. Adventitial *transfer constant* was 0.129 min^−1^ before therapy and 0.099 min^−1^ after 1-year treatment. There was subtle gradation of color in adventitial zone (arrows). Reprint with permission from [91].

Cornily et al. in their animal experiments on abdominal aorta acquired from 10 rabbits (7 atherosclerotic and 3 control rabbits) showed a significant enhancement in the atherosclerotic group following injection of MRI contrast agent [90]. A strong correlation was found between plaque enhancement and plaque neovessel density using contrast-enhanced MRI. Kerwin et al. used DCE-MRI to perform quantitative analysis of the adventitia in patients with carotid artery disease [95]. In 25 patients with carotid endarterectomy specimens, DCE-MRI showed a significant correlation between adventitial transfer constant with the amount of neovasculature and macrophage (*P* < 0.05). The average transfer constant was significantly higher in patients with severe carotid stenosis than that in patients with moderate disease (0.155 ± 0.045 min^−1^ versus 0.122 ± 0.029 min^−1^, *P* < 0.01). The transfer of the contrast agent to the adventitia depends on the vasa vasorum; therefore, adventitial transfer constant is regarded as an indicator to reflect the extent of the vasa vasorum [91, 95]. This is further validated by a recent study by Dong et al. who studied 28 patients using DCE-MRI at both baseline and 1-year follow-up [91]. After 12 months of lipid therapy, a significant reduction was observed in mean adventitial transfer constant when compared to the baseline (0.067 min^−1^  ±0.028 versus 0.085 min^−1^  ±0.037, *P* = 0.02) (Figure 5). The adventitial transfer constant was reduced due to the reduction of volume, permeability, or both in neovessels arising from the adventitia. These findings suggest that DCE-MRI could be used as an effective method for the assessment of therapeutic effects on carotid artery atherosclerosis.

In summary, studies have confirmed that contrast-enhanced MRI, in particular, DCE-MRI, provides unique advantages to visualize components of atherosclerotic plaques and identify vulnerable plaques in vivo imaging [96–101]. There is a good correlation between atherosclerotic plaque enhancement on MRI imaging and neovessel density of the plaques and signs of inflammation in the plaques in patients with acute coronary syndrome [102–104]. Thus, contrast-enhanced MRI is considered to be a reliable non-invasive tool to detect and characterize features of vulnerable plaques in vivo.

## 3. Summary and Conclusion

Rapid technological advances in imaging modalities have augmented the ability of these imaging techniques to detect and characterize cardiovascular disease, offering information beyond the traditional assessment of lumen changes. Micro-CT demonstrates excellent anatomical details of vasa vasorum due to its superior resolution; however, it is only limited to animal or human cadaver experiments because of its limited field of view which is an inherent disadvantage of the micro-CT scanner. IVUS and OCT are an intravascular imaging modality with unique characteristics of providing superior information on the vascular lumen, plaque components, and vasa vasorum. In particular, OCT has superior resolution which enables it to provide detailed structural information such as different types of plaques. However, both imaging techniques are invasive and are restricted to limited clinical centres. Furthermore, methodology of image acquisition and interpretation needs to be standardized.

CEUS and DCE-MRI both provide important insight into plaque components and microvascular features which are associated with plaque vulnerability; thus, these two non-invasive imaging modalities serve as an effective means for assessing risk status of atherosclerotic plaques and monitoring therapeutic outcomes of atherosclerotic vascular disease. Large prospective clinical trials are needed to demonstrate how these modalities improve patient care by modifying patient management and outcome.
